# COVID-19 pandemic related long-term chronic stress on the prevalence of depression and anxiety in the general population

**DOI:** 10.1186/s12888-021-03385-x

**Published:** 2021-07-28

**Authors:** Tian Qi, Ting Hu, Qi-Qi Ge, Xiao-Na Zhou, Jia-Mei Li, Chun-Lei Jiang, Wei Wang

**Affiliations:** 1 The Battalion 3 of Cadet Brigade, School of Basic Medicine, Navy Medical University, 800 Xiangyin Road, Shanghai, China; 2 Department of Stress Medicine, Faculty of Psychology, Navy Medical University, 800 Xiangyin Road, Shanghai, China

**Keywords:** COVID-19, Long-term stress, Depression, Anxiety

## Abstract

**Background:**

The COVID-19 pandemic has lasted for more than 1 year, causing far-reaching and unprecedented changes in almost all aspects of society. This study aimed to evaluate the long-term consequences of the COVID-19 pandemic on depression and anxiety, and explore the factors associated with it.

**Methods:**

A cross-sectional study using an online survey was conducted to assess mental health problems from February 2 to February 9, 2021 by using patient health questionnaire-9 (PHQ-9) and generalized anxiety disorder-7 (GAD-7). The insomnia severity index (ISI), demographic data and COVID-19 related variables were measured by a self-designed questionnaire. The factors associated with depressive and anxiety symptoms were identified by Pearson chi-square test and binary logistic regression analysis.

**Results:**

In the study that 1171 participants enrolled, the overall prevalence of depressive and anxiety symptoms among general people was 22.6 and 21.4% respectively in the present study. Living alone was a potential risk factor for depressive symptoms, while regular exercises was a potential protective factor. The prevalence of depressive and anxiety symptoms was significantly associated with the severity of insomnia symptoms and the negative feelings about pandemic.

**Conclusion:**

COVID-19 pandemic- related chronic stress has brought about profound impacts on long-term mental health in the general population. The level of insomnia and a negative attitude towards the pandemic are significantly correlated with unfavorable mental health. However, we failed to found a significant association of age and gender with the mental health symptoms, although they were recognized as well-established risk factors during the outbreak by some other studies. This discrepancy may be because the acute and chronic effects of the pandemic are influenced by different factors, which reminds that more attention should be paid to the intrinsic psychological factors and physical reactions towards COVID-19.

**Supplementary Information:**

The online version contains supplementary material available at 10.1186/s12888-021-03385-x.

## Introduction

The 2019 coronavirus disease (COVID-19) pandemic has become a global health threat, according to the latest data from the World Health Organization (WHO), over 110 million people have been infected worldwide by February 21, 2021, with more than 2.4 million deaths reported [1]. In the early stage of the COVID-19 pandemic, people were highly exposed to acute biopsychosocial stressors generated by the pandemic, and many studies reported a high prevalence of psychological distress in health care workers (HCWs) and general populations [2–5]. One study with more than 1200 respondents from almost 200 cities in China during January and February 2020 showed that about 54% respondents rated the psychological impact of the COVID-19 moderately or severely; about 29% respondents reported moderate to severe anxiety symptoms; and less than 17% reported moderate to severe depressive symptoms [3]. Another study demonstrated a high prevalence of depression (50.7%), anxiety (44.7%) and stress-related symptoms (73.4%) in HCWs [2]. Wang et al. [3] reported that 16.5% individuals in the general population experienced moderate to severe depressive symptoms, and 28.8% experienced moderate to severe anxiety symptoms. A meta-analysis [6] reported that the prevalence of pandemic-related depression and anxiety in the general population was 33.7% and 31.9 respectively. In HCWs [7], anxiety was assessed with a pooled prevalence of 23.2% in 12 studies, and depression was assessed with a pooled prevalence of 22.8% in 10 studies.

Additionally, variables such as occupation, education background and gender were found to have impact on the symptoms of anxiety and depression during the pandemic [8]. The female gender, younger age, higher education background and students were significantly associated with more negative psychological effects of COVID-19 and higher levels of stress, anxiety, and depression [3]. There was also a significant correlation between psychological outcomes with specific physical symptoms, like myalgias, dizziness, and coryza [9]. During the outbreak, the public were encouraged to stay at home. The strict lockdown for about 3 months imposed greater challenges on children and adolescents in terms of psychological problems and psychiatric disorders [10]. Decreased social relations, to a great extent, increased the psychological negative feelings of the whole family [11, 12].

In the year when the coronavirus continues to ravage the world, China has adopted lots of unprecedented measures to control the COVID-19 transmission, including the suspension of public transportation, the closing of public spaces, close management of communities, and isolation and care for infected people and suspected cases [13]. Although China has basically achieved the success of pandemic prevention and control, outbreaks caused by local cases or imported cases still exist in different provinces during the whole year. To curb the spread of the coronavirus during the ox Spring Festival holidays, China issued a plan to reduce mass gatherings and strengthen the pandemic control by taking such measures as advocating off-peak travel and encouraging people to celebrate in place. These responses to this severe public health emergency affected and changed our ways of socializing, working, studying, and living [14]. These drastic changes put general people under extra stressful conditions. Chronic stress, especially psychosocial stressors in humans, is a well-known risk factor for the development of depression and anxiety [15–19], which can lead to a variety of emotional and physical problems and can decrease a person’s ability to function at both work and at home [20, 21]. Early psychological first aid can play the key role in alleviating the mental health symptoms [22, 23], but the remote intervene couldn’t be implemented on large scale limited by the economic conditions.

Unlike severe acute respiratory syndrome (SARS), the COVID-19 pandemic is likely to become a global pandemic with unknown duration. The research on long-term outcomes among SARS survivors reported the main psychiatric morbidity was post-traumatic stress disorder (PTSD) and the prevalence was up to 25%, while 15.6% of the patients had depressive disorders [24]. As for the general public, studies showed an increase in anxiety and depressive symptoms during the epidemic [25, 26]. However, a prospective cohort study on college students indicated the subjects’ anxiety levels gradually reduced over time in latter stage of SARS epidemic [27]. As a significant psychological stressor, COVID-19 has brought about tremendous impact on every facet of individuals’ lives. People will be affected by the crushing wave for a long time, both psychologically and physiologically [28]. However, the long-term effects of the pandemic on psychology are still unknown and unpredictable.

Accordingly, we aim to evaluate the long-term consequences caused by COVID-19 pandemic on mental health status and identity factors associated with it among the general population in China preliminarily, knowing that a better understanding about the psychological changes of the public in such situations will help develop effective mental health measures, and provide guiding clues to encounter another situation like this COVID-19 pandemic in the future.

## Methods

### Study design and setting

We conducted this cross-sectional study using an online survey to assess mental health problems from February 2 to February 9, 2021, which was the 1-year time point after the peak of COVID-19 outbreak. Respondents were recruited to participate in the online survey through the Wenjuanxing platform (https://www.wjx.cn/app/survey.aspx). We recruited 20 volunteers aged from 18 to 60 for sharing the link address of questionnaire by WeChat (which is similar with Facebook) and also shared it to their eldership by short message. Adults aged 18 years and over were eligible to participate. In total,1300 participants took part in the survey. After removing the data of participants with incomplete (or complete all questionnaires less than 120 s) or unreasonable questionnaires, 1171 participants from different provinces were included in the analysis. Of these,494 (42.2%) participants were women and 677(57.8%) were men.

### Measurements

#### Depression

The patient health questionnaire-9 (PHQ-9) was used in the present study [29]. The PHQ-9 was based on the diagnostic criteria for depression from the Diagnostic and Statistical Manual of Mental Disorders, 4th Edition (DSM-IV) [30]. The response option were:0= “not at all”,1 = “several days”, 2= “more than half the days” and 3= “nearly every day”. A two-week recall period was used. The total score ranged from 0 to 27, with a higher score indicating greater self-reported depression. The PHQ-9 scale score was divided into five categories: no (0–4), mild (5–9), moderate (10–14), moderately severe (15–19), and severe (20–27). A total score of ≥10 indicates possible major depression, with a sensitivity of 80% and specificity of 92% [31, 32]. The psychometric properties of the PHQ-9 have been previously confirmed in Chinese population [33]. In the present study, the Cronbach’s alpha coefficient of the PHQ-9 was 0.910.

#### Anxiety

The generalized anxiety disorder-7 (GAD-7) was used to measure the severity of self-reported anxiety [34]. The response options were: 0= “not at all”, 1 = “several days”, 2= “more than half the days”, and 3= “nearly every day”. A two-week recall period was used. The GAD-7 scale score was divided into four categories: no (0–4), mild (5–9), moderate (10–14), and severe (15–21). The total score ranged from 0 to 21, with a higher score indicating greater self-reported anxiety. For the GAD-7, a total score of ≥10 indicated possible anxiety, with the optional point for sensitivity (89%) and specificity (82%) [35, 36]. In the present study, the Cronbach’s alpha coefficient of the GAD-7 was 0.941.

#### Insomnia

The insomnia severity index (ISI) addresses the nature, severity, and impact of insomnia during the previous 2 weeks [37, 38]. The items assess the severity of the difficulties in falling asleep, maintaining sleep and early morning awakening, the degree of (dis) satisfaction with the current sleep pattern, interference with daily functioning, noticeable impairment due to sleep problems by others, and worry or distress about sleep problems. Each item is rated on a 5-point Likert scale, yielding a total score ranging from 0 to 28, interpreted as follows: no clinical insomnia (0–7); subthreshold insomnia (8–14); moderate insomnia (15–21); and severe insomnia (22–28). In the present study, the Cronbach’s alpha coefficient of the ISI was 0.904.

#### Social support

Social support was measured by the Social Support Rating Scale (SSRS) designed by Xiao [39], measuring three dimensions of social support: subjective support (4 items), objective support (3 items) and support-seeking behavior (3 items). SSRS contains 10 items with a total score of 60, and the score increases with growing social support. A score of less than 33 represents low levels of social support, a score of 33–45 represents normal levels of social support, and scores between 45 and 60 represent high levels of social support. The SSRS has been used with a wide range of Chinese populations due to its high reliability and validity, with 2-month test–retest reliability of 0.92.

#### General information and independent variables

Sociodemographic data were collected including age, gender, education level, employment status, marital status and income. We also collected the life experiences data, including separation with spouse, solitary status and exercise habits. Besides, the data related to pandemic were collected including contacting history, negative or positive feelings about pandemic, isolation history, psychological consulting, etc., during COVID-19 pandemic period by self-reported questions. Most of the independent variables were bi-variate variables (Yes/No).

### Statistical analysis

Categorical data were described using counts (n, %). Descriptive statistics were performed using χ^2^ tests and post-hoc analysis with the Bonferroni correction (Table S1, S2). The variables that significantly differed in the χ^2^ analyses were included in Binary logistic regression model to test significant associations. All statistical analyses were conducted using Statistical Package for the Social Sciences (SPSS) (version 22.0), with *p*-values < 0.05 indicating statistically significant. Only respondents providing full data were included in the analysis, and imputation or other substitution methods were not used.

## Results

### The prevalence of depressive and anxiety symptoms

**Fig. 1:**
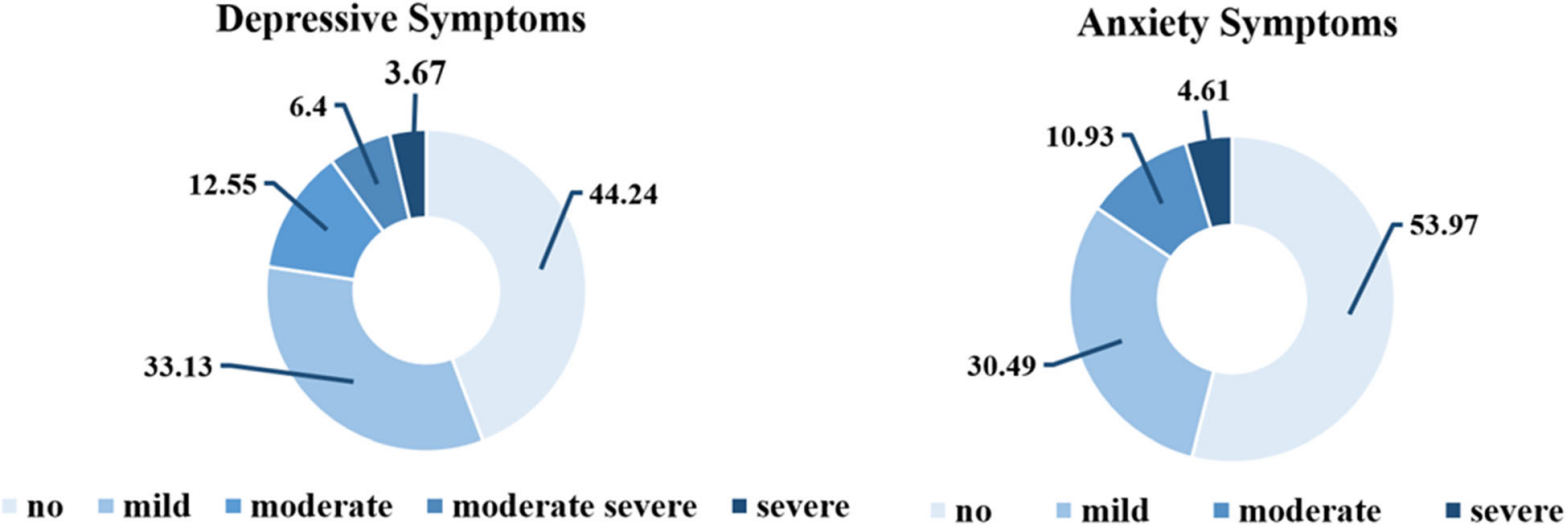
Distribution of the mental disorders in the total population

A total of 1300 participants from different provinces in China were invited to complete the survey, and 1171 effective questionnaires were collected, with a 90.1% response rate, including 494 men (42.19%) and 677 women (57.81%). The results are shown in Table 1. Among this final sample, the mean age of the male and female participants was 31.66 and 34.61 years old respectively. The median (interquartile range) scores for PHQ-9 were 5 (2–9) and 4 (1–7) for GAD-7. The overall prevalence of depressive and anxiety symptoms among our participants were 22.6% (PHQ-9 score ≥ 10) and 21.4% (GAD-7 score ≥ 10), respectively. According to the grades of symptoms of previous researches [31, 36], the distribution of the depressive and anxiety symptoms in the total population is shown in Fig. 1. The effects of demographic variables and COVID-19 related questions on the prevalence of depressive and anxiety symptoms were analyzed by chi-square test. The results showed the association between depression, anxiety, demographic variables, and COVID-19 related questions. The participants with depressive symptoms were more likely to live alone, less likely to exercises regularly compared with participants with non-depressive symptoms. Similarly, participants in the anxiety symptoms group were also more likely to live alone and less likely to exercises regularly compared with those in the non-anxiety symptoms group. However, the distribution of depressive and anxiety symptoms was not different in terms of gender, age, living situation, education, marital status, and the contacting history. The post hoc analyses with Bonferroni correction showed that participants with depressive and anxiety symptoms were more likely to be low-income household.

**Table 1:** Association between depression, anxiety and demographic variables

	n (%)	Depressive symptoms	Non-depressive symptoms	*p*	Anxiety symptoms	Non-anxiety symptoms	*p*
		*n* = 265(%)	*n* = 906(%)		*n* = 251(%)	*n* = 920(%)	
Demographic data							
Gender (female)	677(57.8)	157(59.2)	520(57.4)	0.592	140(55.8)	537(58.4)	0.461
Age							
18–25	336(28.7)	71(26.8)	265(29.2)	0.099	56(22.3)	280(30.4)	0.075
26–30	208(17.8)	57(21.5)	151(16.7)		55(21.9)	153(16.6)	
31–40	364(31.1)	89(33.6)	275(30.4)		84(33.5)	280(30.4)	
41–50	185(15.8)	37(14.0)	148(16.3)		41(16.3)	144(15.7)	
51 above	78(6.7)	11(4.2)	67(7.4)		15(6.0)	63(6.8)	
Living situation (Shanghai)	370(31.6)	82(30.9)	288(31.8)	0.795	82(32.7)	288(31.3)	0.680
Household Income							
Low^a^	406(34.7)	113(42.6)	293(32.3)	0.004	110(43.8)	296(32.2)	0.001
Middle	648(55.3)	134(50.6)	514(56.7)		124(49.4)	524(57.0)	
High	117(10.0)	18(6.8)	99(10.9)		17(6.8)	100(10.9)	
Education							
High school	93(7.9)	19(7.2)	74(8.2)	0.255	21(8.4)	72(7.8)	0.759
Junior college	200(17.1)	58(21.9)	142(15.7)		46(18.3)	154(16.7)	
Bachelor	664(56.7)	142(53.6)	522(57.6)		133(53.0)	531(57.7)	
Master	156(13.3)	33(12.5)	123(13.6)		37(14.7)	119(12.9)	
Doctor	58(5.0)	13(4.9)	45(5.0)		14(5.6)	44(4.8)	
Marital status							
Single	342(29.2)	84(31.7)	258(28.5)	0.781	73(29.1)	269(29.2)	0.445
Romantic partner	133(11.4)	30(11.3)	103(11.4)		22(8.8)	111(12.1)	
Married	658(56.2)	143(54.0)	515(56.8)		149(59.4)	509(55.3)	
Widow or divorced	38(3.2)	8(3.0)	30(3.3)		7(2.8)	31(3.4)	
Living alone	277(23.7)	92(34.7)	185(20.4)	< 0.001	80(31.9)	197(21.4)	< 0.001
Exercise habits	382(32.6)	63(23.8)	319(35.2)	< 0.001	63(25.1)	319(34.7)	0.004

^a^Significant after Bonferroni correction

### Association between depression, anxiety, insomnia and COVID-19 related questions

Compared with individuals with no and mild insomnia symptoms, the depressive and anxiety symptoms were more common in individuals who were inflicted with moderate and severe insomnia (*p* < 0.001). Meanwhile, the depressive and anxiety symptoms tended to be more common in individuals with higher scores of insomnia symptoms on mild level than those in individuals without insomnia symptoms. The severity of the depressive and anxiety symptoms was also significantly different between individuals who thought of the pandemic as having brought about negative effects on their life and those who had no or mild such feelings (*p* < 0.001) (Table 2).

**Table 2:** Association between depression, anxiety, insomnia and COVID-19 related questions

	n(%)	Depressive symptoms	Non-depressive symptoms	*p*	Anxiety symptoms	Non-anxiety symptoms	*p*
		*n =* 265(%)	*n =* 906(%)		*n =* 251(%)	*n =* 920(%)	
Contacting history	45(3.8)	10(3.8)	35(3.9)	0.947	12(4.8)	33(3.6)	0.383
Insomnia							
No^a^	722(61.7)	67(25.3)	655(72.3)	< 0.001	64(25.5)	658(71.5)	< 0.001
Mild^a^	326(27.8)	107(40.4)	219(24.2)		99(39.4)	227(24.7)	
Moderate	96(8.2)	66(24.9)	30(3.3)		63(25.1)	33(3.6)	
Severe	27(2.3)	25(9.4)	2(0.2)		25(10.0)	2(0.2)	
Negative feelings about pandemic							
No^a^	227(19.4)	22(8.3)	205(22.6)	< 0.001	27(10.8)	200(21.7)	< 0.001
Little^a^	246(21.0)	42(15.8)	204(22.5)		41(16.3)	205(22.3)	
Mild^a^	350(29.9)	76(28.7)	274(30.2)		69(27.5)	281(30.5)	
Moderate	198(16.9)	59(22.3)	139(15.3)		57(22.7)	141(15.3)	
Severe	150(12.8)	66(24.9)	84(9.3)		57(22.7)	93(10.1)	

^a^Significant after Bonferroni correction

### Correlations between demographic variables, insomnia, COVID-19 related questions and depressive and anxiety symptoms

Logistic regression analysis was performed to determine the factors associated with depressive symptoms (Hosmer and Lemeshow test, χ^2^ = 7.290, *p* = 0.506) (Table 3), and anxiety symptoms (Hosmer and Lemeshow test, χ^2^ = 7.081, *p* = 0.528) (Table 4). Multicollinearity was assessed among the co-variates with Variance Inflation Factor (VIF) and it was evident that no multicollinearity existed (mean VIF = 1.038, maximum VIF = 1.061, minimum VIF = 1.025). Living status (living alone) (OR = 0.559, CI = [0.390, 0.804]), negative feelings about the pandemic (thought that the negative effect of the pandemic was mild (OR = 1.923, CI = [1.081, 3.420]),moderate (OR = 3.205, CI = [1.747, 5.883]) and severe (OR = 5.010, CI = [2.679, 9.368]), regular exercise (OR = 0.587, CI = [0.402, 0.858]) and insomnia symptoms (mild: OR = 3.982, CI = [2.795, 5.673]; moderate: OR = 19.578, CI = [11.613, 33.006]; severe: OR = 97.099, CI = [21.636, 435.765]) were found to be significantly associated depressive symptoms. However, we found that only the negative feeling about the pandemic and the severity of insomnia symptoms were significantly associated with anxiety symptoms. The former was categorized as the moderate negative feeling (OR = 2.288, CI = [1.290, 4.056]) and the severe negative feeling (OR = 2.676, CI = [1.471, 4.866]), and the latter was categorized as mild insomnia symptoms (OR = 3.908, CI = [2.731, 5.592]), moderate insomnia symptoms (OR = 17.886, CI = [10.770, 29.705]) and severe insomnia symptoms (OR = 98.650, CI = [22.530, 431.955]). Moreover, no potential protective factors for anxiety symptoms were found in the present study.

**Table 3:** Correlations between demographic variables, insomnia, COVID-19 related questions and Depressive symptoms

Variable	B	S.E.	O.R.	95 C.I.	VIF
Household Income (ref: Low)					1.029
Middle	−0.133	0.177	0.875	0.618, 1.239	
High	−0.425	0.325	0.654	0.346, 1.236	
Living alone	−0.581	0.185	0.559	0.390, 0.804	1.025
Exercise habits	− 0.532	0.193	0.587	0.402, 0.858	1.032
Insomnia (ref: No)					1.061
Mild	1.382	0.181	3.982	2.795, 5.673	
Moderate	2.974	0.266	19.578	11.613, 33.006	
Severe	4.576	0.766	97.099	21.636, 435.765	
Negative feelings about pandemic (ref: No)					1.042
Little	0.422	0.320	1.524	0.814, 2.854	
Mild	0.654	0.294	1.923	1.081, 3.420	
Moderate	1.165	0.310	3.205	1.747, 5.883	
Severe	1.611	0.319	5.010	2.679, 9.368

**Table 4:** Correlations between demographic variables, insomnia, COVID-19 related questions and Anxiety symptoms

Variable	B	S.E.	O.R.	95 C.I.	VIF
Household Income (ref: Low)					1.029
Middle	−0.260	0.176	0.771	0.546, 1.090	
High	− 0.533	0.327	0.587	0.309, 1.114	
Living alone	−0.294	0.187	0.745	0.516, 1.077	1.025
Exercise habits	−0.363	0.191	0.695	0.479, 1.011	1.032
Insomnia (ref: No)					1.061
Mild	1.363	0.183	3.908	2.731, 5.592	
Moderate	2.884	0.259	17.886	10.770, 29.705	
Severe	4.592	0.753	98.650	22.530, 431.955	
Negative feelings about pandemic (ref: No)					1.042
Little	0.157	0.303	1.171	0.647, 2.118	
Mild	0.240	0.277	1.271	0.738, 2.188	
Moderate	0.827	0.292	2.288	1.290, 4.056	
Severe	0.984	0.305	2.676	1.471, 4.866	

## Discussion

About 1 year after the COVID-19 pandemic first outbreak in China, we conducted a cross-sectional study involving 1174 Chinese adults and found that the prevalence of depression and anxiety was 22.6 and 21.4% respectively. The inclusion scores for depression and anxiety were PHQ-9 ≥ 10 and GAD-7 ≥ 10 respectively. When we aligned our standards with PHQ-9 ≥ 5 and GAD-7 ≥ 5, the prevalence of depression and anxiety improved to 44.2 and 54.0% respectively. Some population surveys in the early phase of COVID-19 investigated the short-term impact of the COVID-19 outbreak on mental health and well-being in the general population, but the results about the incidence of depression and anxiety were not consistent between the studies, ranging from 9 to 51% for depression, and from 10 to 45% for anxiety, probably due to the different measuring tools and cut-off value settings. A meta-analysis reported an aggregate prevalence of 31.5% (24.2–39.2%) for depression and 29.8% (21.5–38.8%) for anxiety in the general population [14]. Another Meta-analysis reported an overall prevalence of 31.9% for anxiety and 33.7% for depression in the general population [6]. Based on the findings obtained from early surveys concerning the impact of the pandemic on mental health of the public, we found no significant change in the prevalence of depression and anxiety as the pandemic continued, indicating that the impact of the pandemic on mental health has persisted and not been attenuated significantly despite the pandemic control. Individuals’ fear and worries generated by SARS were likely subsided as the treatment methods were identified [27], while vaccines and treatments of COVID-19 remained inconclusive until now. It is reasonable to concern about the long-term effects of COVID-19 on mental health.

According to the WHO, the total number of people living with depression in the world is 322 million worldwide. The estimated proportion of the global population with depression is about 4.4% in 2015. From the region, age and gender dimensions, the report also found that depression is more common among females than that in males (5.1% vs. 3.6%) [40]. Huang et al. reported that the lifetime prevalence of depression in China is 6.9% and the 12-month prevalence rate is 3.6% [41]. The number of women with depression is approximately twice that of men (65% vs. 35%). Previous reports also indicated that the female gender was a risk factor for depression and anxiety [40], but this phenomenon was not found in our research. Our speculation that result is based on the following considerations. On the one hand, previous studies have demonstrated that females are more likely to develop anxiety and panic than males [42, 43]. In the early phase of the COVID-19 pandemic, people were exposed to fear of the unknown virus, and females were more easily affected by such fear. On the other hand, with the increasing understanding about and more effective control of the pandemic in China, such fear began subsiding gradually, and the pandemic-related chronic stress was more reflected in the socioeconomic aspect due to social isolations such as controlling mobility and gathering of people and limiting social activities between people [44], all of which may cause the increasing chronic stress in males. Study on college students showed higher prevalence of depression among male students and were mostly interpreted as the social isolation limited relaxation and stress release [45]. In regarding with the younger demographic structure of the study, the result with no significant difference in gender is likely due to the increased incidence of chronic stress in young males who inclined to regulate mental health by outdoor activities. There is, in addition, in Chinese socioeconomic-cultural circumstances, males are generally considered as breadwinners of the family [46] and men were suffered more financial pressure. The male breadwinners have to explore outside to earn, which might increase the infection risk and also rise the trauma reexperience about pandemic. Thus, males are more probably to suffer psychological trauma such as depression and anxiety than females.

Emerging evidence indicates that the mental health impacts will be large and long-lasting for a long time, especially in under-resourced contexts and disadvantaged populations [47]. Similarly, our study found the group with low household income had higher prevalence in depression and anxiety. Meanwhile, we speculated that the cognition reconstruction of individual caused by COVID-19 was an important aspect that affects individual mental health. Our data showed that the depression and anxiety scores are high in individuals who thought of the pandemic as having a negative impact on their life. Positive coping skills have been reported as a resilient psychological mechanism versus mental disease [48, 49]. Research showed positive religious coping is vital in reducing anxiety and depression among HCWs amid the pandemic [50], implying that negative feelings towards pandemic may form a potential risk on mental health. It was reported that poverty-related stress remained a direct predictor of higher depressive symptoms [51], which is consistent with our results. Although there is no specific explanation for how low annual incomes trigger depressive and anxious symptoms, some studies reported that poor social support and negative coping could mediate the risk effect of poverty-related stress [52]. So far, there is no study to determine the relationship between low incomes and individual psychological cognition coping with COVID-19, which warrants further study. In our study, we found there was significant correlation between living alone and social support (Table S3). During the pandemic when government advocated social isolation, people inevitably reduced social interactions and living alone may result in the low level of social support. It was reported that individuals with less social support more likely suffered from depression [48, 52, 53], which was consistent with our findings that people who live alone have lower social support and more symptoms of depression and anxiety.

Although the pandemic has caused worldwide impacts on public life, including entertainment, education, work and other aspects, individuals can also take initiatives to relieve their symptoms of psychological distress. In our study, individuals who maintained regular exercise exhibited fewer and less severe symptoms of depression and anxiety, which is consistent with previous studies [54, 55]. People who exercised daily presented fewer somatic symptoms, lower stress levels and more normal sleep than individuals who did not exercise [56]. Besides, ample evidence showed there is a correlation between insomnia, depression and anxiety [57–59]. Our result showed a significant difference in depressive or anxiety symptoms between individuals with different grades of insomnia, demonstrating that individuals with moderate to severe insomnia were more inclined to get depressive and anxiety symptoms than those with mild or no insomnia. Our ongoing research is to further explore the mechanism underlying the association between insomnia and depression and anxiety.

## Limitation

There are several limitations in this study. First, although network questionnaire survey could obtain a large number of samples based on the WeChat program, it has its own problems of reliability and validity which cannot be ignored. The self-reported levels of psychological impact, anxiety, depression and stress may not always be aligned with objective assessment by mental health professionals. Because of the difference of online habits between youth and older adults, the demographics of the survey were skewed towards younger population. Thus, our data may only represent the population aged 18–40, which is also a limitation of our study. For the assessment of mental health status of people over 50 years old, paper and pen surveys or face-to-face interviews may be more appropriate. Second, we were unable to collect the follow-up data during the pandemic due to the social isolation policy, which may not accurately and completely reflect the past histories because of the recall bias. Third, the survey failed to collect the data about pre-existing stress, anxiety and depression of the participants, knowing that some respondents with high baseline scores of stress, anxiety and depression may report scores over standardized scales, so that the score may not simply be attributable to the COVID-19 pandemic. Fourth, we did not collect detailed information about exposure history, which might influence current psychological status. Finally, the correlations could not be used for causal inferences due to the synchronicity of independent and dependent variable measurements. This is the biggest disadvantage of the cross-sectional study.

## Conclusion

To the best of our knowledge, this is the first study addressing the mental health in a Chinese general population after more than 1 year of the COVID-19 pandemic. We found that COVID-19 pandemic related long-term chronic stress has profound impacts on the long-term mental health of the general population. The proportion of people with symptoms of depression, anxiety and insomnia remains high even 1 year after the COVID-19 outbreak in China. Insomnia was significantly correlated with depression and anxiety. These findings highlight the need for more clinical attention to the insomnia symptom of the public, as well as the need for adjusting the public coping mode to the pandemic. Our study also revealed that a low household income and living alone were potential risk factors affecting the mental health, which is often ignored in the information wave of the pandemic and various kinds of social news. Above all, the results of this study may assist government agencies and healthcare professionals in safeguarding the public psychological well-being in the context of COVID-19.

## Supplementary Information

**Additional file 1: Table S1**. Post hoc analysis on depressive model. **Table S2**. Post hoc analysis on anxiety model. **Table S3**. Correlation between social support, living alone and mental health.

## Data Availability

The datasets analyzed and materials used in this study are available from the corresponding author (Wei Wang) on reasonable request.
