# Supplementary material for: COVID-19 pandemic related long-term chronic stress on the prevalence of depression and anxiety in the general population
Source: BMC Psychiatry. 2021 Jul 28;21:380. doi: 10.1186/s12888-021-03385-x (PMC8316891; doi:10.1186/s12888-021-03385-x)
Supplement: Supplementary file 1 — Additional file 1: Table S1. Post hoc analysis on depressive model. Table S2. Post hoc analysis on anxiety model. Table S3. Correlation between social support, living alone and mental health. [file 12888_2021_3385_MOESM1_ESM.docx]

Table S.1 Post hoc analysis on depressive model

|  | I | Low | | Middle | | II | No | | Mild | | Moderate | | III | No | | Little | | Mild | | Moderate | |
| --- | --- | --- | --- | --- | --- | --- | --- | --- | --- | --- | --- | --- | --- | --- | --- | --- | --- | --- | --- | --- | --- |
|  |  | χ^2^ | *p* | χ^2^ | *p* |  | χ^2^ | *p* | χ^2^ | *p* | χ^2^ | *p* |  | χ^2^ | *p* | χ^2^ | *p* | χ^2^ | *p* | χ^2^ | *p* |
| Household Income(I) |  |  | |  | |  |  | |  | |  | |  |  | |  | |  | |  | |
| Low |  |  | |  | |  |  | |  | |  | |  |  | |  | |  | |  | |
| Middle |  | 7.119 | 0.024^*^ |  | |  |  | |  | |  | |  |  | |  | |  | |  | |
| High |  | 7.496 | 0.018^*^ | 1.745 | 0.561 |  |  | |  | |  | |  |  | |  | |  | |  | |
| Insomnia (II) |  |  | |  | |  |  | |  | |  | |  |  | |  | |  | |  | |
| No |  |  | |  | |  |  | |  | |  | |  |  | |  | |  | |  | |
| Mild |  |  | |  | |  | 89.899 | <0.001^***^ |  | |  | |  |  | |  | |  | |  | |
| Moderate |  |  | |  | |  | 220.099 | <0.001^***^ | 39.575 | <0.001^***^ |  | |  |  | |  | |  | |  | |
| Severe |  |  | |  | |  | 167.669 | <0.001^***^ | 38.051 | <0.001^***^ | 6.224 | 0.078 |  |  | |  | |  | |  | |
| Negative feelings about pandemic (III) |  |  | |  | |  |  | |  | |  | |  |  | |  | |  | |  | |
| No |  |  | |  | |  |  | |  | |  | |  |  | |  | |  | |  | |
| Little |  |  | |  | |  |  | |  | |  | |  | 5.498 | 0.190 |  | |  | |  | |
| Mild |  |  | |  | |  |  | |  | |  | |  | 14.116 | 0.002^**^ | 1.960 | 1.615 |  | |  | |
| Moderate |  |  | |  | |  |  | |  | |  | |  | 27.714 | <0.001^***^ | 10.108 | 0.015^*^ | 4.451 | 0.349 |  | |
| Severe |  |  | |  | |  |  | |  | |  | |  | 59.413 | <0.001^***^ | 34.062 | <0.001^***^ | 25.645 | <0.001^***^ | 7.479 | 0.062 |

*: *p<*0.05, **: *p<*0.01, ***: *p<*0.001; All *p* -values were *Bonferroni* corrected.

Table S.2 Post hoc analysis on anxiety model

|  | I | Low | | Middle | | II | No | | | Mild | | Moderate | | III | No | | | Little | | | | | Mild | | | Moderate | | |
| --- | --- | --- | --- | --- | --- | --- | --- | --- | --- | --- | --- | --- | --- | --- | --- | --- | --- | --- | --- | --- | --- | --- | --- | --- | --- | --- | --- | --- |
|  |  | χ^2^ | *p* | χ^2^ | *p* |  | χ^2^ | *p* | | χ^2^ | *p* | χ^2^ | *p* |  | χ^2^ | *p* | χ^2^ | | *p* | | | χ^2^ | | *p* | | χ^2^ | *p* | |
| Household Income(I) |  |  | |  | |  |  | | |  | |  | |  |  | | |  | | | | |  | | |  | | |
| Low |  |  | |  | |  |  | | |  | |  | |  |  | | |  | | | | |  | | |  | | |
| Middle |  | 9.152 | 0.006^**^ |  | |  |  | | |  | |  | |  |  | | |  | | | | |  | | |  | | |
| High |  | 7.797 | 0.015^*^ | 1.398 | 0.711 |  |  | | |  | |  | |  |  | | |  | | | | |  | | |  | | |
| Insomnia(II) |  |  | |  | |  |  | | |  | |  | |  |  | | |  | | | | |  | | |  | | |
| No |  |  | |  | |  |  | | |  | |  | |  |  | | |  | | | | |  | | |  | | |
| Mild |  |  | |  | |  | 79.071 | <0.001^***^ | |  | |  | |  |  | | |  | | | | |  | | |  | | |
| Moderate |  |  | |  | |  | 208.150 | | <0.001^***^ | 38.976 | <0.001^***^ |  | |  |  | | |  | | | | |  | | |  | | |
| Severe |  |  | |  | |  | 174.258 | | <0.001^***^ | 42.366 | <0.001^***^ | 7.528 | 0.036^*^ |  |  | | |  | | | | |  | | |  | | |
| Negative feelings about pandemic(III) |  |  | |  | |  |  | | |  | |  | |  |  | | |  | | | | |  | | |  | | |
| No |  |  | |  | |  |  | | |  | |  | |  |  | | |  | | | | |  | | |  | | |
| Little |  |  | |  | |  |  | | |  | |  | |  | 2.184 | 1.394 | |  | | | | |  | | |  | | |
| Mild |  |  | |  | |  |  | | |  | |  | |  | 6.071 | 0.137 | | 0.892 | | 3.451 | | |  | | |  | | |
| Moderate |  |  | |  | |  |  | | |  | |  | |  | 19.032 | <0.001^***^ | | 9.371 | | 0.022^*^ | | | 5.880 | | 0.153 |  | | |
| Severe |  |  | |  | |  |  | | |  | |  | |  | 35.545 | <0.001^***^ | | 22.772 | | | <0.001^***^ | | 18.626 | | <0.001^***^ | 3.288 | | 0.698 |

*: *p<*0.05, **: *p<*0.01, ***: *p<*0.001; All *p* -values were *Bonferroni* corrected;

Table S.3 Correlation between social support, living alone and mental health.

|  | Living alone | Social support | Depression | Anxiety |
| --- | --- | --- | --- | --- |
| Living alone | 1.00 |  | | |
| Social support | 0.282^**^ |  |  |  |
| Depression | -0.141^**^ | -0.340^**^ |  |  |
| Anxiety | -0.101^**^ | -0.278^**^ | 0.613^**^ | 1.00 |

^**^P<0.05, Living alone was measured as bi-variate variable. A score of 1 represents “living alone” while a score of 2 means “not living alone”. Social support was measured by SSRS (Social Support Rating Scale) as consecutive variate. Depression and anxiety were measured by PHQ-9 (Patient Health Questionnaire-9) and GAD-7 (Generalized Anxiety Disorder-7) as bi-variate variable, respectively. The inclusion scores for depression and anxiety were PHQ-9≥10 and GAD-7≥10 respectively. The correlation between social support and living alone, depression or anxiety was analyzed by point biserial correlation analysis. The correlation between depression, living alone and anxiety were analyzed by Correlation analysis of chi square test. The biserial correlation coefficients and Phi coefficients were presented in the table S.3.
